# Factors Affecting Postoperative Lung Functions in Patients Undergoing Lobectomy for Non-Small Cell Lung Cancer

**DOI:** 10.3390/medicina58081021

**Published:** 2022-07-29

**Authors:** Soo-Jin Lee, Hyo-Yeong Ahn, Jong-Hwan Park, Jeong-Su Cho

**Affiliations:** 1 Department of Thoracic and Cardiovascular Surgery, Medical Research Institute, Pusan National University Hospital, Busan 49241, Korea; lsj31514401@gmail.com (S.-J.L.); drmozart@pusan.ac.kr (J.-S.C.); 2 Department of Health Convergence Medicine, Biomedical Research Institute, Pusan National University Hospital, Busan 49241, Korea

**Keywords:** lobectomy, body mass index, lung function test

## Abstract

*Background and Objectives*: The estimation of lung function impairment after pulmonary lobectomy for primary non-small cell lung cancer (NSCLC) has been of great interest since the reduction of respiratory function might severely affect a patient’s quality of life. The perioperative factors that may have an influence on widening the gap between the postoperative measured lung function and predicted postoperative lung function were our greatest concern. We aimed to analyze the perioperative patient factors that may influence postoperative lung function in patients undergoing pulmonary lobectomy. *Materials and Methods*: A retrospective study was conducted using the medical records of 199 patients who underwent lobectomy for lung cancer between July 2017 and May 2020. After comparing the achieved postoperative forced expiratory volume in 1 s (FEV1) and predicted postoperative (ppo) FEV1, patients were divided into two groups: group A (*n* = 127), who had preserved pulmonary lung function; and group B (*n* = 72), who had decreased pulmonary lung function. Primary endpoints included location of pulmonary resection, preoperative performance status, body mass index (BMI) on admission, total muscle area, and muscle index. *Results* In group A, the proportion of normal weighted patients was significantly higher than that in group B (67.7% vs. 47.2%, *p* = 0.003). Conversely, the proportion of overweight patients was significantly higher in group B than in group A (47.2% vs. 28.3%, *p* = 0.003). Group B had a significantly high incidence of upper lobe resection (*p* = 0.012). The mean total muscle area in group A was higher than that in group B, but the difference was not statistically significant. *Conclusions*: A greater decrease in postoperative lung function than in ppo FEV1 was associated with BMI and the location of pulmonary resection in patients who underwent lobectomy. Postoperative physiologic changes due to high BMI and the resection of upper lobes need to be discussed to prevent postoperative morbidities.

## 1. Introduction

Pulmonary lobectomy is the standard operative treatment for primary non-small cell lung cancer (NSCLC) [1]. The resection of lung parenchyma decreases lung function in patients, and it can reveal poor respiratory function, which reduces quality of life [2]. To select suitable candidates for lobectomy, vital capacity (VC) and forced expiratory volume in 1 s(FEV1) are the most commonly used representative indexes of lung functions [3]. Predicted postoperative lung function is the mainstay for assessing perioperative risk after lobectomy, and it is assessed by the number of segments removed [4].

However, measured lung function is not always within the predicted postoperative lung function [5]. The difference between the postoperative measured lung function and predicted postoperative lung function prompted us to analyze the perioperative factors that may have a negative influence on postoperative lung function in patients undergoing lobectomy.

## 2. Materials and Methods

### 2.1. Statement of Ethics

This clinical study is purely observational and thus does not need registration. The study was conducted with medical records that cannot indicate a patient’s personal information or be recognized. This study was approved by the Institutional Review Board of Pusan National University Hospital. (IRB No. 2203-012-112) The requirement for informed consent was waived because the analysis was retrospectively performed based on electronic patient records.

### 2.2. Patients

**Figure 1:**
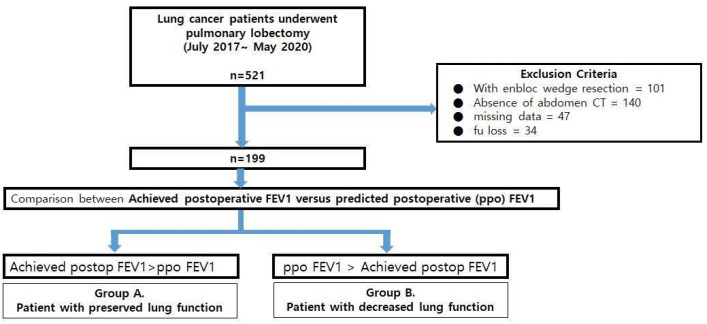
The flow chart of the study.

This study was conducted using the medical records of 521 patients who underwent pulmonary lobectomy for lung cancer at Pusan National University Hospital (PNUH) between July 2017 and May 2020. Patients who underwent additional wedge resection or en bloc wedge resection (*n* = 101) were excluded. Patients who did not have preoperative abdominal CT images (a prerequisite for analyzing muscle mass index and one of the perioperative factors [*n* = 140]) or missing data (*n* = 81) were also excluded. Central lesions that obstructed more than 2 subbronchial bronchi were also one of the exclusion criteria of this study, but none of them was found. Finally, 199 patients were enrolled in the analysis (Figure 1).

### 2.3. Comparison between Predicted Postoperative Lung Function after Lobectomy(ppo FEV1%) and Measured Lung Function after Lobectomy (Achieved Postoperative FEV1%: apoFEV1%)

A representative index of expected lung function after lobectomy in this study was ppo FEV1 using a segmental method as follows: ppo FEV1(%) = preoperative FEV1 × remaining segments/19 [6]. Apo FEV1 was representative of lung function after lobectomy, and it was conducted from 4 to 5 months after surgery. Comparing these two indexes, patients were divided into two groups: group A(*n* = 127), apoFEV1 larger than ppo FEV1; and group B (*n* = 72), apoFEV1 smaller than ppo FEV (Figure 1).

Analyzed perioperative factors are divided into three categories: first, preoperative factors including age, sex, smoking habit, clinical stage, comorbidities, body mass index (BMI) on admission, total muscle area, muscle index, lung volume measured with an automated lung image analysis tool (Thoracic VCAR, GE Healthcare, Chicago, IL, USA), and performance status; second, intraoperative factors including resected location of the pulmonary lobe, approach modality such as video-assisted thoracoscopic surgery or conventional thoracotomy, conversion to thoracotomy, and operative time; lastly, postoperative factors included total intensive unit days, degree of pain, length of hospital stay, pulmonary, cardiovascular, and infectious complications, and operative mortality within 30 days.

### 2.4. Statistical Analysis

The baseline characteristics between two groups were analyzed, and perioperative factors were compared with an independent t-test or Wilcoxon rank-sum test for continuous variables and Fisher’s test for categorical variables. Multivariate analysis using a logistic regression test was conducted. A *p* value less than 0.05 was considered to be statistically significant. R software (version 4.0.1; R Development Core Team, Vienna, Austria) was used for all statistical analyses.

## 3. Results

### 3.1. Baseline Patients’ Characteristics for Group A and Group B

Comparative results of patient characteristics between Groups A and B are summarized in Table 1. The patients were divided into two groups, with group A containing 127 patients and group B containing 72 patients. There were no statistical differences in baseline characteristics between the two groups.

### 3.2. Comparison of Perioperative Factors between Groups A and B

The resected location of pulmonary lobe and BMI were significantly different between the two groups. Among 48 patients who underwent left upper lobectomy, 23 (18.3%) showed more preserved pulmonary function than expected (group A), and 25 (34.7%) were categorized as group B. In 52 patients who underwent right upper lobectomy, 27 (21.4 %) categorized as group A and 25 (34.7%) were categorized as group B (*p* = 0.002). Conversely, patients who underwent other lobes (RML, RLL, and LLL) showed a higher proportion of patients in group B than in group A [group A vs. group b (*n*, %); 7 (5.6) vs. 3 (4.2); 40 (31.7) vs. 13 (18.1); 29 (23.0) vs. 6 (8.3)].

Normal weight patients (*n* = 120) had a larger proportion in group A (67.7%) than in group B (47.2%). Overweight patients (*n* = 70) were divided into group A, 28.3%, and group B, 47.2%. Among the other nine patients, four patients (2%) were underweight and five patients (2.5%) were obese (Table 2).

The mean values of total muscle area and muscle mass index were higher in group A compared with group B, but the difference was not statistically significant (Table 2). In addition, performance status and other factors including approach modality, whether the operation converted to open procedure, operative time, incidence of postoperative complications, short-term mortality, and length of ICU and hospital stay did not show significant differences between the two groups (*p* > 0.05) (Table 3).

In the multivariate analysis, resection of LUL and RUL and overweight were identified as independent factors influencing decreased lung function after lobectomy (*p* < 0.05) (Table 4).

## 4. Discussion

To evaluate a proper lobectomy candidate and establish a surgical plan, a pulmonary function test (PFT) is routinely performed before surgery for patients who are expected to undergo pulmonary lobectomy. Ppo FEV1 is one of the measurements generally used to select suitable candidates for lobectomy, since its spirometric predicted values correlate with postoperative FEV1 and FVC. 

**Figure 2:**
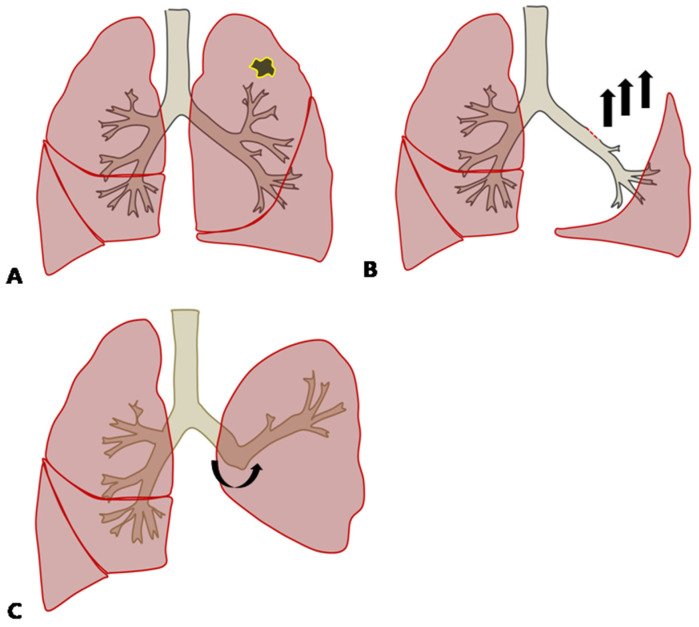
(**A**) In cases with lung cancer in the left upper lobe, left upper lobectomy (LUL) might be performed. (**B**) During LUL, the inferior pulmonary ligament is dissected, which induces the remaining left lower lobe to expand and move upward. (**C**) The remaining left lower bronchus is angulated upward after LUL.

The analysis showed that upper lobectomy and BMI were independent factors which led to greater reduction in postoperative lung function than by ppo FEV1 in the early postoperative phase. Although the mean resected lobar volume was the highest in LUL, ppo FEV1 was supposed to be the calculation of all the segments that would remain after resection, which meant that there was no regard for the resected lobar volume. This could be explained by the compensatory response after left upper lobectomy, in which the inferior pulmonary ligament was divided to enhance the expansion of the lower lobe. The remaining left lower bronchus would be angulated upward, which causes bronchial distortion and reduces pulmonary ventilation and the perfusion of the remaining lung after left upper lobectomy (Figure 2) [7]. Although both LUL and RUL were the statistically significant factors affecting postoperative lung function, the odds ratio of LUL was 1.25 times larger than that for RUL on multivariate analysis, meaning that LUL has the greatest influence of any location. Since the volume of LUL is the largest [8], it could be explained by more dramatic physiologic changes compared with after right upper lobectomy, which causes more severe ventilation and perfusion disturbance.

Additionally, BMI affects postoperative lung function more than ppo FEV1 in the early postoperative phase. A large amount of abdominal fat in obese patients increases intra-abdominal pressure, which might prevent the diaphragm from moving down during deep breathing, decreasing the functional residual capacity and lung compliance. Thus, increased intraabdominal pressure results in increased respiratory demand [9,10] and affects physiologic changes in the inflation of the remaining lung in obese patients. However, there was no further difference between the obese and underweight groups or the normal and underweight groups due to the limited number of patients.

Recent studies suggest that preoperative pulmonary rehabilitation has a significant impact on improving exercise performance in high-risk patients undergoing lobectomy for NSCLC [11,12]. Therefore, preoperative pulmonary rehabilitation might be necessary in high-risk cases to decrease ppo FEV1 after lobectomy, such as upper lobectomy and high BMI.

However, this study was conducted retrospectively in a single center, and there might be a selection bias for the patients enrolled. Additionally the time of the evaluation of postoperative pulmonary function ranged from 4 to 5 months but was not identical among the patients included in this study, and this might have affected the results. Explaining the diaphragm movement and high BMI as being associated with abdominal obesity may give rise to a question since high BMI does not always represent abdominal obesity. Thus, the value achieved from the direct measurement of abdominal fat from abdominal CT, which represents abdominal obesity, could be used as an effective index representing excessive abdominal fat. 

Furthermore, CT-based simulation might be required to analyze how the remaining lung inflates and remodels after lobectomy to investigate the different compensatory lung responses after lobectomy.

## 5. Conclusions

The greatest gap between postoperative FEV1 compared with ppo FEV1 was associated with BMI and the location of pulmonary resection in patients who underwent lobectomy. Due to physiologic changes in patients with high BMI undergoing upper lobectomy, postoperative pulmonary rehabilitations may have clinical implications for postoperative lung function.

## Figures and Tables

**Table 1:** Patient characteristics of group A vs. group B.

Characteristics		Overall (*n* = 199)	Group A. (*n* = 127)	Group B. (*n* = 72)	*p* Value
Age (mean (SD))		67.26 (7.12)	67.28 (7.20)	67.21 (7.04)	0.943
Sex	Male	126 (63.3)	86 (67.7)	40 (55.6)	0.119
	Female	73 (36.7)	41 (32.3)	32 (44.4)	
Smoking habit	Never	93 (46.7)	53 (41.7)	40 (55.6)	0.170
	Ex smoker	82 (41.2)	57 (44.9)	25 (34.7)	
	current	24 (12.1)	17 (13.4)	7 (9.7)	
clinical stage(%)	IA1	23 (11.6)	13 (10.2)	10 (13.9)	0.291
	IA2	32 (16.1)	18 (14.2)	14 (19.4)	
	IA3	36 (18.1)	28 (22.0)	8 (11.1)	
	IB	43 (21.6)	24 (18.9)	19 (26.4)	
	IIA	20 (10.1)	15 (11.8)	5 (6.9)	
	IIB	20 (10.1)	11 (8.7)	9 (12.5)	
	IIIA	23 (11.6)	17 (13.4)	6 (8.3)	
	IIIB	2 (1.0)	1 (0.8)	1 (1.4)	
Comorbidity	COPD(%)	13 (6.5)	9 (7.1)	4 (5.6)	0.903
	CAOD(%)	8 (4.0)	6 (4.7)	2 (2.8)	0.767
	CVS(%)	28 (14.1)	18 (14.2)	10 (13.9)	1.000
	CHF(%)	1 (0.5)	1 (0.8)	0 (0.0)	1.000
	DM(%)	40 (20.1)	28 (22.0)	12 (16.7)	0.468
	CRF(%)	6 (3.0)	5 (3.9)	1 (1.4)	0.563

COPD: chronic obstructive pulmonary disease), CAOD: coronary artery occlusive disease, CVS: cerebrovascular stroke, CHF: congestive heart failure, DM: Diabetes Mellitus, CRF: chronic renal failure.

**Table 2:** Perioperative factors with statistical significance between Group A and Group B.

Patient Factors		Overall (*n* = 199)	Group A. (*n* = 127)	Group B. (*n* = 72)	*p* Value
Resected location (%)	RUL	52 (26.3)	27 (21.4)	25 (34.7)	0.002
	RML	10 (5.1)	7 (5.6)	3 (4.2)	
	RLL	53 (26.8)	40 (31.7)	13 (18.1)	
	LUL	48 (24.2)	23 (18.3)	25 (34.7)	
	LLL	35 (17.7)	29 (23.0)	6 (8.3)	
BMI (%)	underweight	4 (2.0)	1 (0.8)	3 (4.2)	0.012
	normal	120 (60.3)	86 (67.7)	34 (47.2)	
	overweight	70 (35.2)	36 (28.3)	34 (47.2)	
	obese	5 (2.5)	4 (3.1)	1 (1.4)	
Total muscle area (mean (SD))		119.34 (26.02)	121.47 (27.03)	115.06 (23.87)	0.320
muscle mass index (mean (SD))		44.45 (9.86)	45.31 (8.84)	42.82 (11.61)	0.363

RUL: right upper lobe, RML: right middle lobe, RLL: right lower lobe, LUL: left upper lobe, LLL: left lower lobe, BMI: body mass index.

**Table 3:** Perioperative factors with statistical insignificance between group A and group B.

Patient Factors		Overall (*n* = 199)	Group A. (*n* = 127)	Group B. (*n* = 72)	*p* Value
performance status (%)	0	192 (96.5)	122 (96.1)	70 (97.2)	0.743
	1	6 (3.0)	4 (3.1)	2 (2.8)	
	2	1 (0.5)	1 (0.8)	0 (0.0)	
Approach modality (%)	VATS	183 (92.0)	118 (92.9)	65 (90.3)	0.700
	open	16 (8.0)	9 (7.1)	7 (9.7)	
conversion to open (%)		1 (0.5)	1 (0.8)	0 (0.0)	1.000
operative time(mean (SD))		3.09 (1.36)	3.06 (1.16)	3.14 (1.67)	0.705
ICU stay (days(%))	none	13 (6.5)	3 (4.2)	3 (4.2)	0.325
	0 ≤ ≤ 1	172 (86.4)	107 (84.3)	65 (90.3)	
	1 < ≤ 2	10 (5.0)	8 (6.3)	2 (2.8)	
	2 < ≤ 4	1 (0.5)	0 (0.0)	1 (1.4)	
	4 < ≤ 5	1 (0.5)	1 (0.8)	0 (0.0)	
	6 < ≤ 8	1 (0.5)	1 (0.8)	0 (0.0)	
	9 < ≤ 16	1 (0.5)	0 (0.0)	1 (1.4)	
Hospital stay (mean (SD))		7.66 (5.19)	7.51 (4.85)	7.93 (5.76)	0.603
postoperative complication	pulmonary	10 (5.0)	7 (5.5)	3 (4.2)	0.936
	cardiovascular	0 (0)	0 (0)	0 (0)	-
	infectious	0 (0)	0 (0)	0 (0)	-
	other	4 (2.0)	2 (1.6)	2 (2.8)	0.956
30-day mortality		10 (5.0)	7 (5.5)	3 (4.2)	0.936

**Table 4:** Perioperative factors with statistical significance between group A and group B by multivariate analysis.

	Overall (*n* = 199)	Group A. (*n* = 127)	Group B. (*n* = 72)	Multivariate Analysis	
				OR [95% CI]	*p* Value
BMI (%)	normal	86 (67.7)	34 (47.2)	Ref.	
	underweight	1 (0.8)	3 (4.2)	9.459 [0.839, 106.693]	0.069
	overweight	36 (28.3)	34 (47.2)	2.278 [1.185, 4.380]	0.014
	obese	4 (3.1)	1 (1.4)	0.659 [0.066, 6.568]	0.722
Location (%)	LLL	29 (23.0)	6 (8.3)	Ref.	
	RUL	27 (21.4)	25 (34.7)	4.691 [1.613, 13.647]	0.005
	RML	7 (5.6)	3 (4.2)	2.067 [0.396, 10.784]	0.389
	RLL	40 (31.7)	13 (18.1)	1.929 [0.629, 5.913]	0.250
	LUL	23 (18.3)	25 (34.7)	5.871 [1.987, 17.345]	0.001

OR: odds ratio, CI: confidence interval, Ref.: reference.

## Data Availability

Not applicable.
